# Henry Steven Greer, MD, FRCPsych, FRANZP

**DOI:** 10.1192/bjb.2022.68

**Published:** 2023-04

**Authors:** Paul Crichton

Formerly Consultant Psychiatrist, Royal Marsden Hospital, London, UK


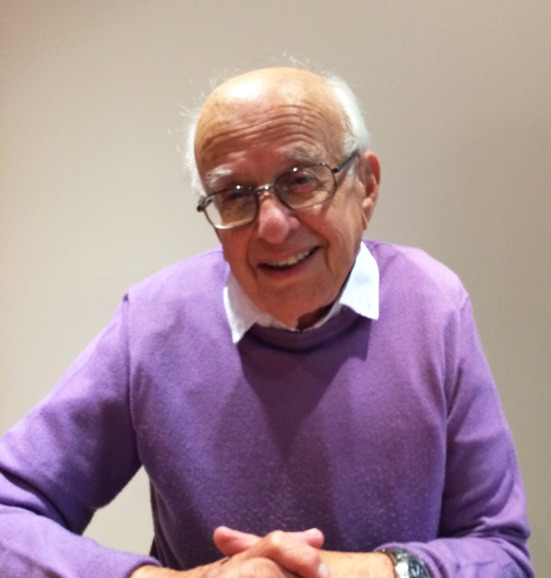


Henry Steven Greer (Steven), who died on 12 March 2022 aged 93, was one of the pioneers in the treatment of the psychological problems affecting people with cancer. He was a leading figure in establishing the field of psycho-oncology in the UK, the treatment of psychological problems afflicting people with cancer. He co-founded the first psycho-oncology department and carried out, with colleagues, some of the first ground-breaking research in the subject. As a consequence, many people with cancer have benefited from his psychological insights and from the type of psychotherapy, based on cognitive–behavioural principles, that he and Stirling Moorey developed to treat the complex and sometimes severe psychological symptoms that may distress people with cancer. Together with his medical colleagues, Keith Pettingale and Dudley Tee, he founded the Faith Courtauld Unit for Human Studies in Cancer at King's College Hospital Medical School, London, and in 1983, co-founded the British Psychosocial Oncology Society. In 1996, he was awarded the Arthur M. Sutherland Award of the International Psycho-Oncology Society in New York for his pioneering work in psycho-oncology.

Steven Greer was born in Vienna on 14 August 1928, the only child of Charles and Carola (née Goldhammer) Gershwint. He was very close to his father, who worked as a general practitioner. Because of the rise in anti-Semitism in Austria, while he was still a child his family emigrated to Australia by ship. At that time, the family name was changed from Gershwint to Greer. After growing up in an affluent part of Vienna he found it difficult living in the Australian outback. He was sent to boarding school where, as a Jewish immigrant, he struggled to fit in.

Steven studied medicine at Adelaide Medical School, South Australia, graduating in 1952 and then worked in general practice. Becoming increasingly interested in psychiatry he came to London in 1957, where he trained in psychiatry at the Maudsley Hospital from 1957 to 1960. While at the Maudsley Hospital he completed an MD thesis on the natural history of neurotic illness. He was impressed by the intellectual rigour of Aubrey Lewis and, whenever he was writing a paper, he thought of Lewis saying over his shoulder ‘When you write that sentence, what is your evidence?’.

After returning briefly to Australia, he came back to London in 1964 to take up an appointment at King's College Hospital Medical School, where, in 1968, he became Reader in Psychological Medicine. In 1986, at the invitation of Professor Tim McElwain, he moved to the Royal Marsden Hospital, where, with his colleague Maggie Watson, he set up a Department of Psychological Medicine. He was especially concerned with ‘fighting spirit’ and its possible effect on the duration of survival of women with breast cancer. The cognitive–behavioural therapy that he evolved with Stirling Moorey was shown in several randomised controlled studies to significantly improve the lives of people with cancer.^1^ This led to the publication in 1989 of the first textbook on the subject in the UK (*Psychological Therapy for Patients with Cancer: A New Approach*), now published as *Oxford Guide to CBT for People with Cancer*.^2^ Steven remained at the Royal Marsden Hospital until he retired. After retirement, he continued to work part-time at St Raphael's Hospice in Cheam, south-west London.

Steven treated his patients with singular respect for their individual needs as well as for their autonomy and dignity. He had remarkable empathy for his patients and a deep understanding of the wide range of psychological reactions to the diagnosis of cancer and how to treat them. He was very supportive of colleagues, always eager to pass on his knowledge and experience. Those who met him outside the medical context would never have been able to guess how eminent he was in his field, so modest was he in his demeanour. He was tolerant and humane, but nonetheless capable of making witty and incisive comments on others.

He was married three times but had no children. He met his third wife, Carol Sells, when they were working at the same hospital, she as an occupational healthcare assistant and he as a psychiatrist. After having been good friends for many years, which Carol Sells attributes to the fact they shared a birthday (although he was 20 years older) they married in 2017.

Steven was a small man, with an open smile. He returned to Austria only once, retained a basic knowledge of German and spoke English with an Australian twang. He enjoyed Australian wine, Mozart's music and English poetry, especially John Keats and Elizabeth Barrett Browning. He remained curious and interested in a wide range of topics, far beyond psychiatry, until the very end of his life. These interests included European history since 1789, animal welfare (he was a vegetarian and involved in several animal welfare organisations), ethology, evolution, and the ethical and philosophical aspects of psychiatry and medicine.

He is survived by his wife Carol Sells.
